# Start Position Strongly Influences Fixation Patterns during Face Processing: Difficulties with Eye Movements as a Measure of Information Use

**DOI:** 10.1371/journal.pone.0031106

**Published:** 2012-02-02

**Authors:** Joseph Arizpe, Dwight J. Kravitz, Galit Yovel, Chris I. Baker

**Affiliations:** 1 Laboratory of Brain and Cognition, National Institute of Mental Health, National Institutes of Health, Bethesda, Maryland, United States of America; 2 Visual Cognition Group, Institute of Cognitive Neuroscience, University College London, London, United Kingdom; 3 Department of Psychology, Tel Aviv University, Tel Aviv, Israel; University of British Columbia, Canada

## Abstract

Fixation patterns are thought to reflect cognitive processing and, thus, index the most informative stimulus features for task performance. During face recognition, initial fixations to the center of the nose have been taken to indicate this location is optimal for information extraction. However, the use of fixations as a marker for information use rests on the assumption that fixation patterns are predominantly determined by stimulus and task, despite the fact that fixations are also influenced by visuo-motor factors. Here, we tested the effect of starting position on fixation patterns during a face recognition task with upright and inverted faces. While we observed differences in fixations between upright and inverted faces, likely reflecting differences in cognitive processing, there was also a strong effect of start position. Over the first five saccades, fixation patterns across start positions were only coarsely similar, with most fixations around the eyes. Importantly, however, the precise fixation pattern was highly dependent on start position with a strong tendency toward facial features furthest from the start position. For example, the often-reported tendency toward the left over right eye was reversed for the left starting position. Further, delayed initial saccades for central versus peripheral start positions suggest greater information processing prior to the initial saccade, highlighting the experimental bias introduced by the commonly used center start position. Finally, the precise effect of face inversion on fixation patterns was also dependent on start position. These results demonstrate the importance of a non-stimulus, non-task factor in determining fixation patterns. The patterns observed likely reflect a complex combination of visuo-motor effects and simple sampling strategies as well as cognitive factors. These different factors are very difficult to tease apart and therefore great caution must be applied when interpreting absolute fixation locations as indicative of information use, particularly at a fine spatial scale.

## Introduction

The location of visual fixations are often assumed to directly reflect the allocation of visual attention [1]. Thus, their spatial and temporal pattern may indicate the regions of a stimulus being processed for use in a particular task and give direct insight into cognitive processes [2], [3], [4], [5]. Consider the following two examples. First, based on fixation locations during face recognition, Hsiao and Cottrell concluded that fixations near the center of the nose were optimal for recognition [6]. Second, Blais and colleagues [7] noted a difference in fixation patterns during face viewing between Asian and Caucasian observers and concluded that this difference reflected the impact of culture on high-level face processing strategies. However, the use of fixations to infer stimulus- and task-dependent visual processing assumes that the specific stimuli and the task are the primary determinants of the fixation pattern rather than, for example, visuomotor factors. Here we tested this assumption in a study of face processing by varying the initial starting position of the eyes relative to the face, a factor which varies both within and across previous studies. If the pattern of fixations is largely determined by the stimulus and the task, this manipulation should have minimal impact on the overall pattern of fixations.

Analyses of fixation patterns have been used extensively in studies of face processing. While much information can be extracted from single fixations to rapidly presented faces, eye movements appear to be functionally useful, with impaired recognition when fixation location is fixed compared to when participants are free to move their eyes [8], [9]. Most eye tracking studies of face perception report the same basic pattern, with the vast majority of fixations falling on internal facial features and a tendency toward the upper part of the face, and in particular the eyes [7], [10], [11], [12], [13], [14]. Further, the specific pattern of fixations observed is modulated by task [15], [16], [17] and face familiarity [10], [11], [14], [18], [19]. Variations in the basic pattern of fixations have been used as evidence for differences in visual processing between identity and expression tasks [15], upright and inverted faces [10] (but see [20]), Asian and Caucasian observers [7], [21], 5- and 7- week old infants [22], own- and other-race faces [23], patient groups and controls (e.g. Autism Spectrum Disorders: [24], [25], [26]) and between conspecific and non-conspecific faces [27]. However, the extent to which measured fixations during facial processing reflect factors other than stimulus and task is unclear.

We were interested in the impact of starting fixation position on the pattern of visual fixations. Most studies of fixation patterns during face perception consist of a series of trials on which individual faces are presented one at a time with sudden onset. The location of fixation at the onset of the faces is typically controlled (e.g. [6], [7], [10], [28]) although in some cases it is relatively unconstrained [26], [27]. In those studies that do fix the start position, the center of the screen, typically corresponding to the center of the upcoming stimulus is commonly used [11], [14], [23], although exactly which part of the face this corresponds to is highly variable and often unclear). However, increasingly, single [19], [20] or multiple off-face starting positions are employed [6], [7], [21], [28]. In studies in which fixation is unconstrained, observers may spontaneously orient to the center of the screen (i.e. the center of the expected face). Whether start position is constrained or not, all of these studies make the implicit assumption that differences in start position have negligible impact on the overall pattern of fixations.

However, there are several ways in which start position may impose significant biases on the pattern of fixations. In particular, the position of the observer's eye relative to the upcoming face defines the initial sampling of that face, which can affect the pattern of fixations throughout a trial. For start positions on the face (e.g. center), initial saccades must of necessity be directed away from the start position, constraining the first observer-generated fixation location. Further, information is sampled at the start position, perhaps making it less likely the participants will return to that position on later fixations. These considerations have led many to adopt initial fixation locations away from the face (e.g. [6]). However, moving the start position off the face doesn't eliminate potential effects of the initial fixation location. For example, participants may be more likely to saccade initially to the nearest high contrast part of the face, or to saccade to the center of gravity of the face stimulus [28], [29], [30]. Even if the first saccade is aimed at the center of the face, the tendency for saccades to undershoot or overshoot [31], [32] may mean that the actual location of the first fixation differs for different start positions, and the location of this initial fixation may influence subsequent fixations [30]. Finally, face processing has been shown to differ with retinotopic position in the periphery [33] and high-level face-selective cortex has been shown to have position information about presented faces [34]. Given all these considerations, it is important to determine the extent to which eye movements are affected by changes in start position if fixations are to be considered a measure of information use.

Here we systematically tested the effect of start position during a face recognition task on both upright and inverted faces. We chose to manipulate face orientation since inversion produces a reduction in face recognition performance [35], reflecting differences in cognitive processing [36], [37], and has also been reported to have a significant effect on fixation patterns [10] (but see [20], [38]). Thus, we can determine the joint impact of both start position and cognitive factors on fixation patterns during face processing. In our study, participants viewed a series of 40 faces during a study phase before being tested on recognition during a later test phase. We found that the pattern of fixations during both the study and test phase was strongly influenced by start position for both upright and inverted faces. There was also a general effect of inversion, with a greater proportion of fixations on the lower part of inverted compared with upright faces. However, the precise effect of inversion also varied as a function of start position. These findings suggest that eye movements to faces are not wholly predicted by stimuli and task, but may also reflect visuo-motor factors or simple sampling strategies. We conclude that caution is needed in interpreting eye movement patterns solely in terms of information use and high-level visual processing strategies.

## Methods

### Ethics Statement

All participants gave written informed consent and were compensated for their participation. The study was approved by the Institutional Review Board of the National Institutes of Health, Bethesda.

### Participants

20 Caucasian participants (12 male), age 20 to 39. Due to time constraints, one participant only completed the study phase of the experiment and therefore 19/20 participants contributed behavioral and test phase data.

### Eye-tracking

We used an EyeLink II headmounted eye-tracker (SR Research, Mississauga, ON, Canada), and sampled pupil centroid at 500 Hz. The default nine point calibration and validation sequences were repeated throughout the experiment. Both eyes were calibrated and validated, but only the eye with the lowest maximum error was recorded for the trials following a particular calibration. Calibration was repeated when maximum error at validation was more than 1°. Before each trial, a drift correction was performed. Default criteria for fixations, blinks, and saccades implemented in the Eyelink system were used.

### Stimuli

**Figure 1:**
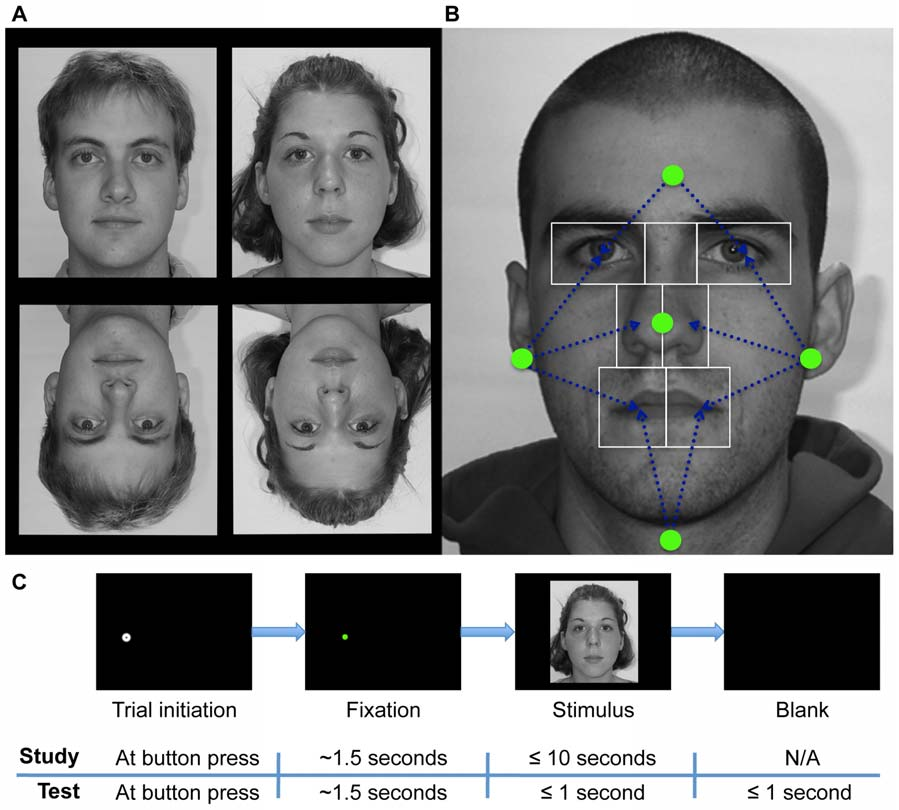
Study design. (**a**) Four example stimuli. Note that all faces were aligned to one another and scaled to be the same size. (**b**) Calculation of start positions. Start positions were determined separately for each face and were defined relative to the face. Left and right start positions were equidistant from centers of the nearest eye, nose and mouth AOIs. Upper and lower start positions were equidistant from the centers of the two eye or two mouth AOIs, respectively. (**c**) Trial sequences in study and test phases. A face was only presented if the participant successfully maintained fixation for a total of 1.5 seconds. After face onset in the study phase, participants were free to study the face for up to 10 seconds and pressed a button to begin the next trial. In the test phase, faces were presented for one second only and participants responded with button presses to indicate whether the face was ‘old’ or ‘new’.

We used 80 grayscale neutral expression face images (40 male) of Caucasians between the ages of 18 and 29 from the Productive Aging Lab Face Database at the University of Texas at Dallas (http://vitallongevity.utdallas.edu/stimuli/facedb/categories/neutral-faces.html) [39]. As stated on the website for the database: “This [database] contains a range of face of all ages which are suitable for use as stimuli in face processing studies. Releases have been signed by the participants we photographed and the faces may be included in publications or in media events.” Each face was scaled to have a 10 degree forehead width at presentation and was rotated to correct any tilt of the head. Images were cropped to remove most of the white background, but not the hair or other external features, and all images were equated for overall luminance (Figure 1a). At presentation, images were centered on a black background. To eliminate any possible stimulus bias as the source of any laterality effects, half of the upright faces were randomly left-right flipped for each participant. Inverted faces were created by simply reflecting each image around the horizontal axis.

### Areas of Interest (AOIs)

For the purposes of analysis and for aspects of our experimental design, rectangular areas-of-interest (AOIs) were drawn for each face around the right and left eyes, bridge of nose (i.e. middle of eye region), right and left half of nose, and right and left half of mouth (Figure 1b, for example) using EyeLink Data Viewer software. AOIs were never visible to participants during the experiment.

### Design

We varied face orientation (upright or inverted) and pre-stimulus fixation location (“start position”) across the trials of an experiment comprised of two phases: study and test. During study, participants observed 40 faces (20 male) in a self-paced manner. At test, participants observed 80 faces (the 40 study phase faces plus 40 new faces) for a limited duration and indicated whether or not they recognized each face as one observed during study (old/new task) (Figure 1c). For each participant, a random half of the faces were inverted in each phase, with the orientation of a given face identical in both phases. The experiment was programmed in Python and interfaced with the eye-tracker.

During study, participants were instructed to study the faces for later recognition and viewed each face for as long as desired up to 10 seconds, self-terminating trials with a button press. The test phase began immediately after the study phase. During test, each face was presented for only one second and then disappeared. Participants indicated by button press whether they recognized the face or not. Participants were instructed to respond within two seconds following stimulus onset, as soon as they thought they knew the answer (Figure 1c).

Start positions were either above, below, right of, left of, or in the center of the internal features of the upcoming face stimulus (e.g. Figure 1b). Coordinates for a start position were calculated uniquely for each face stimulus to be equidistant from all of the nearest internal facial features. For right and left start positions, the unique coordinate that was equidistant from the centers of the nearest eye, nearest half-nose, and nearest half-mouth AOI was calculated numerically for each face. Upper start positions were equidistant from the center of the two eye AOIs, and the lower start positions were equidistant from the two half-mouth AOIs. Distances from the upper and lower start positions to their respective AOI centers were constrained to be the mean of the of the right and left start position distances from their respective AOI centers. The center start position was at the midpoint between the two half-nose AOI centers (Figure 1b).

Before stimulus onset, participants fixated at the start position, indicated by a standard Eyelink II calibration target (0.17° diameter black circle overlaid on a 0.75° diameter white circle) on the black screen. Participants initiated the trial by pressing a button while looking at the fixation target. In this period, a drift correction was performed. A colored dot (0.5° diameter) remained after drift correction, and the stimulus appeared only after a participant had fixated at the dot for an accumulated total of 1500 ms. This ensured that drift correction and fixation were stable prior to stimulus onset. If more than 1500 ms of fixation away from the start position accumulated before the trial could be initiated, drift correction was repeated. A fixation was considered off the start position if it landed more than 0.5° from the center of the dot. Dot color changed successively from red to yellow to green in order to signal to the participant that a maintained fixation was successfully detected at the start position.

In both the study and test phases, there were equal proportions of trials of each combination of levels of the factors of face orientation, face gender, and start position. The particular subset of faces that were in the study phase and also that were inverted was randomized across participants. For a given face, orientation was identical during the study and test phases, but start position varied. Half the faces were presented with the same start position at study and test and for the other half, the start position on the other side of the face was used (e.g. left to right start position between study and test; upper to lower between study and test).

### Analyses

#### Software

Fixation and AOI data were obtained through EyeLink Data Viewer software by SR Research. Subsequent analyses on these data and behavioral data from the test phase were performed with Matlab (The MathWorks, Inc., Natick, MA, USA). ANOVAs were performed in SPSS (IBM, Somers, NY).

#### Behavior

We assessed participants' discrimination performance and reaction time on the old/new recognition task in the test phase. d′ was computed for discrimination performance for each participant, broken down by face orientation and start position. Reaction times for correct trials, broken down by face orientation and start position, were averaged across trials for each participant, and analyses were performed using these values.

#### AOI Analyses

We assessed the relative frequencies of fixations across the AOIs as a function of our experimental manipulations. Given the variable numbers of fixations across trials and across participants, only the first five fixations of each trial were included in the analyses. Relative frequency was calculated for each AOI as the number of actual fixations divided by the total number of possible fixations. ANOVAs on relative frequencies excluded the relative frequency value for the region outside of the AOIs.

#### Spatial Density Analyses

We mapped the spatial density of fixations as a function of our experimental manipulations. Each fixation was plotted with equal density and spatial extent, and so fixations were not weighted by the fixation duration. Fixations beyond the fifth fixation were excluded from the analysis to ensure equal data size across trials. To ensure that summation of fixation maps across different face trials produced spatially meaningful density maps, fixation maps for individual faces were first aligned to a common reference frame using simple translations only. This reference frame was defined by the internal facial features. Specifically, the alignment minimized the sum of the squared differences between the center of the AOIs for each face and the average centers of the AOIs across all 80 faces. Within this common reference frame, fixations were then plotted as Gaussian densities with a mean of 0 and a standard deviation of 0.3° of visual angle in both the x and y dimensions. These density plots were then averaged across trials and across participants. A small proportion of analyzed fixations (<1.6% during study, <0.25% during test) fell outside of the bounds of the stimulus image region (i.e. onto the black background). To ensure equal numbers of fixations in the analyses these fixations were translated to the image edge nearest to the veridical fixation position. The resulting maps show the spatial fixation densities, using a color scale from zero to the maximum density value observed, with zero being transparent. All maps within a single figure contain the same total number of fixations and so are scaled the same to allow for direct comparison. For the same reason, equivalent plots for upright and inverted faces are scaled the same.

#### Spatial Density Contrasts

1) Difference Maps. In order to view differences in the spatial fixation density between two conditions, a pixel-wise subtraction between two spatial density maps was performed for each participant and then averaged across participants. For contrasts between upright and inverted faces, the spatial density map for inverted faces was flipped and aligned with the spatial density map for upright faces before the subtraction.

2) Statistical Maps. In order to produce maps of statistically significant differences in the spatial density map contrasts, a Monte Carlo permutation test was performed on fixation locations between the contrasted conditions. A Monte Carlo permutation test (also called an approximate permutation test or a random permutation test) is a standard, accurate and robust method of performing a significance test on data that is not known to have a parametric (e.g. normal) distribution of values, such as our data. Our statistical analysis is based on methods applied to the analysis of functional brain imaging data [40] and similar to that used in a prior study of eye tracking [41].

The null hypothesis in the Monte Carlo permutation tests was that the distributions of fixation locations of each ordinal fixation (i.e. fixation 1, fixation 2 etc.) were the same between the contrasted conditions (e.g. fixation 1 in upright versus inverted trials, or fixation 3 in right start position versus left). Thus, exchangeability of fixation locations between the given contrasted conditions was assumed only for fixations of the same ordinal value in the sequence of five fixations per trial. 10,400 resampling iterations were performed for each statistical map. For each iteration, locations of fixations were resampled for each individual participant according to the assumed exchangeability, then a new resampled spatial density contrast was produced. These resampled maps were then averaged across participants to produce 10400 group difference maps, the distribution of which was used to determine significance. Maps of p-values were computed pixel-wise based on the number of corresponding pixels in the resampling iterations that were greater than a given positively valued pixel (i.e. where condition 1 had a greater density) in the true spatial density contrast and that were less than a given negatively valued pixel (i.e. condition 2 greater) in the true spatial density contrast. The maps were thresholded at a pixel significance of p<0.01.

For eye-tracking data, our statistical analysis has advantages over other methods of performing significance tests on contrasted fixation maps. A pixel-wise t-test is inappropriate because fixation density data across participants does not approximate a normal distribution at each pixel of a heatmap. Pixel-wise non-parametric tests could create a large multiple comparisons problem, which grows as the pixel resolution of heatmaps grow. In our analysis, fixation locations are exchanged rather than pixels; therefore, increasing the resolution at which heatmaps are displayed does not exacerbate the multiple comparisons problem. Our analysis is an alternative to a Random Field Theory approach, which has been implemented recently by Caldara and colleagues in a free Matlab toolbox called iMap [42].

3) Cluster Corrections for Multiple Comparisons on Statistical Maps. In order to reduce the chance of false positives in our statistical maps due to multiple comparisons, we implemented a nonparametric cluster correction. This correction is based on principles that have been applied to the analysis of functional brain imaging data [40], but, to our knowledge, is novel in the eye-tracking literature. For each analysis, statistical maps were produced for 2600 of the 10400 resampled maps that had resulted from the permutation test. For each of the resulting statistical maps, the size (in pixels) of the largest cluster of p<0.01significance was recorded. Thus a distribution of the maximum cluster size across the iterations of the permutation test was obtained. The size of each cluster in the statistical map of the true data was then compared to the maximum cluster size distribution just obtained. The cluster threshold was set to be p<0.05; therefore, any significant clusters of the true data smaller than the top 5% of the maximum cluster size distribution were eliminated from the statistical map.

## Results

### Discrimination

**Figure 2:**
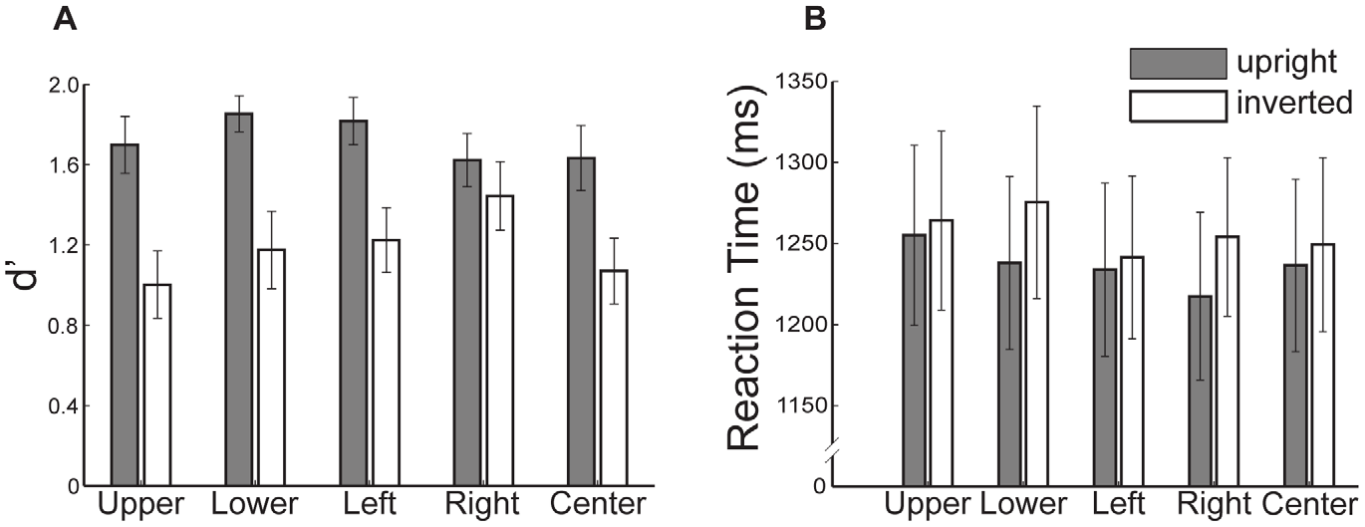
Effects of face inversion on recognition. (**a**) Face recognition, measured by d′, was significantly greater for upright than inverted faces. (**b**) Reaction time also showed an effect of inversion, with longer reaction times for inverted compared to upright faces. Error bars indicate the between-subjects standard error.

Consistent with prior reports we observed a face inversion effect on discrimination scores (d′). A two-way ANOVA, with Orientation (upright, inverted) and Start Position (left, right, center, up, down) as within-subject factors revealed a significant main effect of Orientation with better discrimination for upright than inverted faces (F(1,18) = 29.42, p<0.001). However, there were no main effects or interactions involving Start Position (p>0.15, Figure 2a). An identical two-way ANOVA on reaction time data also revealed a significant main effect of Orientation (F(1,18) = 8.45, p<0.01; Figure 2b) and no main effects or interactions involving Start Position (p>0.14). Further, during study, participants viewed inverted faces longer than upright faces (mean viewing time: upright, 6300 ms, inverted, 6636 ms, t = 3.69, p<0.002).

### Fixation Patterns: Upright Faces

We focus first on eye tracking data for upright faces during the study phase before considering the impact of inversion on the pattern of fixations, and the effect of experiment phase (study versus test).

#### Average Fixations Collapsed Across Start Position Show a Tendency Toward the Left Eye

**Figure 3:**
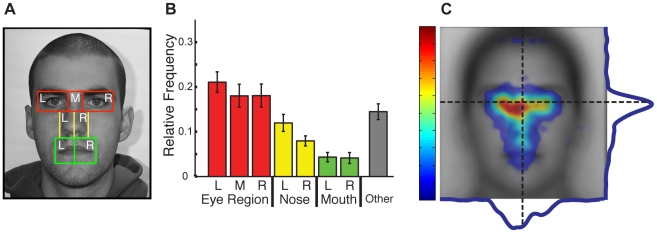
Distribution of fixations for upright faces averaged across start positions. (**a**) Example of AOIs for one face. AOIs could be divided into three separate feature regions: eye (red), nose (yellow), and mouth (green). ‘L’, ‘M’ (eye region only), and ‘R’ refer to the left, middle, and right, respectively, of the facial feature regions. (**b**) Relative frequencies of fixations across AOIs for the first five fixations revealed more fixations to the eye region compared with the nose and mouth regions. Error bars indicate the between-subjects standard error. (**c**) Spatial density and profile plots for the first five fixations showing more fixations to the eye region with a tendency toward the left side of the face. The face plotted beneath the spatial density plot is the average of all faces after alignment. Fixations are plotted as Gaussian densities summed across trials and participants. Fixation density is indicated using a colorscale from zero to the maximum density value observed, with zero being transparent. Profile plots to the right and below the spatial density map are summations of the spatial densities across each dimension. The vertical dotted line indicates the midline of the average face. The horizontal dotted line indicates the vertical position of the center of the eyes.

In order to establish that our data agreed with prior studies (e.g. [6], [7], [12]) in which the effect of start position was not considered, we first analyzed the eye tracking data by pooling across start positions. In both AOI and spatial density analyses we observed the expected tendency toward the upper part of the face (Figure 3). A one-way ANOVA on the relative frequency of fixations (Figure 3b) revealed a significant effect of AOI (F(6,114) = 10.82, p<0.001). Post-hoc t-tests revealed significantly higher relative frequency of fixations for each of the three eye-region AOIs than either of the mouth AOIs (all Bonferroni corrected p<0.015). This is also clear in the spatial density maps where the peak fixation density is just below the eyes and falls off rapidly toward the lower part of the face (Figure 3c). Further, consistent with prior reports (e.g. [6], [43]), there appears to be a tendency toward the left side of the face with a higher relative frequency of fixations for the left than right eye and for the left than right nose. In terms of the spatial density of fixations, the peak of the distribution across participants was shifted to the left of the midline of the face.

While these analyses reveal a similar pattern of fixations to prior studies, we observed a significant effect of start position. In the following section, we break down the results by start position.

#### Fixation Patterns Are Dependent on Start Position

**Figure 4:**
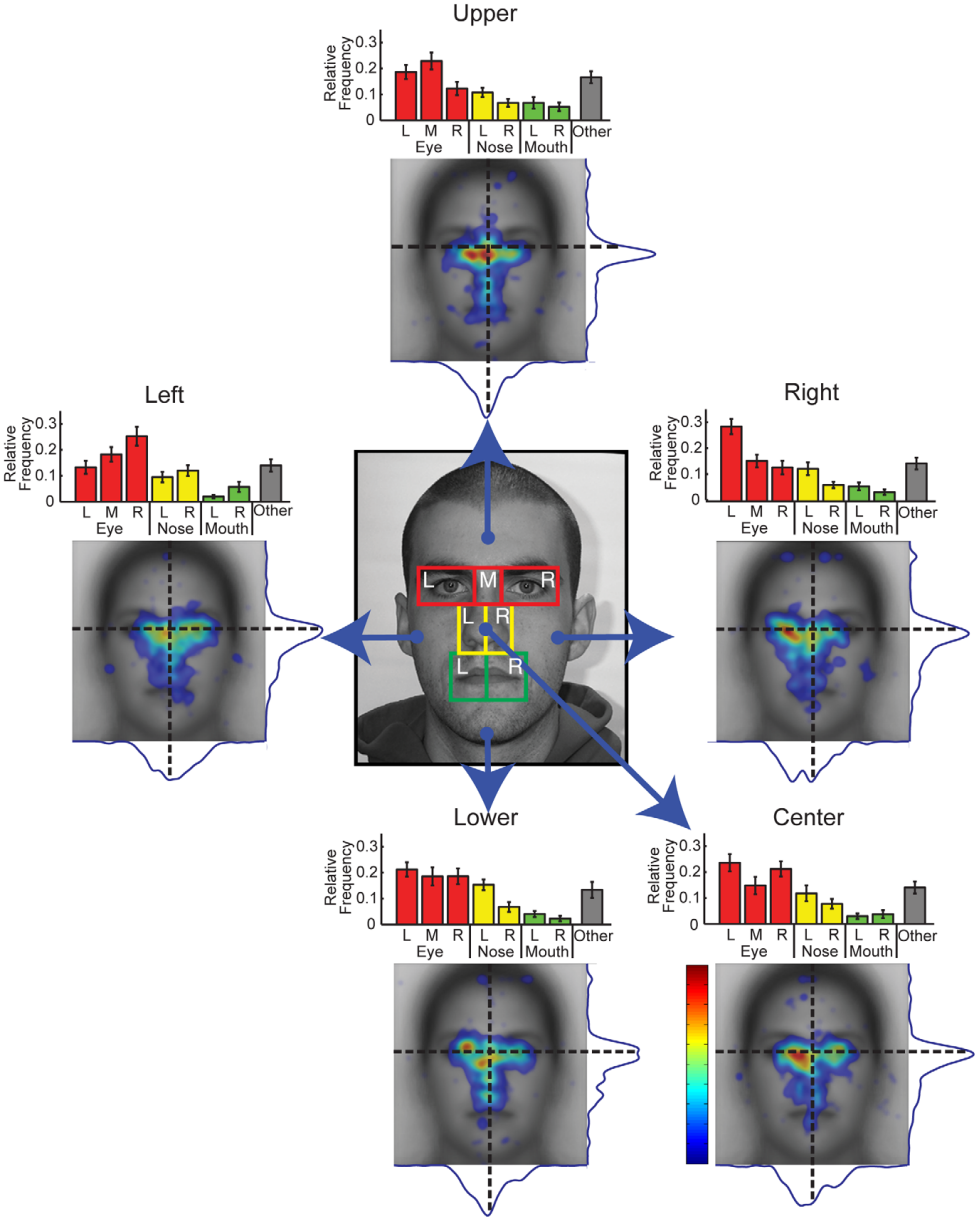
Impact of start position on distribution of fixations for upright faces. AOI, spatial density, and profile plots reveal a strong effect of start position on the distribution of fixations. For example, the overall tendency to one side of the face varies across start positions and switches from the left side of the face for the right start position to the right side of the face for the left start position. Fixation density in the heatmaps is indicated using a colorscale from zero to the maximum density value observed across the five heatmaps, with zero being transparent. Error bars indicate the between-subjects standard error.

Both AOI and spatial density analyses revealed striking differences in fixation patterns as a function of start position (Figure 4). At a coarse level, data from each of the five start positions showed some similarities, with a general tendency for fixations to fall toward the eye region over other parts of the face. However, the specific distribution of fixations across the eyes and other internal features varied substantially. A two-way repeated-measures ANOVA on the relative frequency of fixations with Start Position and AOI (R Eye, M Eye, L Eye, R Nose, L Nose, R Mouth, L Mouth) as factors revealed a significant interaction between start position and AOI (F(24,456) = 5.29, p<0.001). This effect persisted across the first few fixations. A sequence of two-way ANOVAs for each ordinal fixation out to the fifth, with AOI and Start Position as within-subject factors, revealed significant interactions between Start Position and AOI for each of the first three ordinal fixations, and a trend for the fifth (First Fixation: F(24,456) = 5.11, p<0.001; Second Fixation: F(24,456) = 4.34, p<0.001; Third Fixation: F(24,456) = 2.499, p<0.013; Fourth Fixation: F(24,456) = 1.46, p>0.17; Fifth Fixation: F(24,456) = 1.725, p<0.083). Thus, the effect of start position persists beyond the initial fixation.

However, it is clear from the spatial density maps (Figure 3) that the peak fixation density does not often correspond to a unique AOI. Thus, the differences revealed in this analysis are subject to our AOI definitions, which may not be ideal. In later sections, we will consider direct contrasts of the fixations patterns in spatial density maps. However, this initial analysis suffices to establish that start position has a significant impact on the pattern of eye movements observed. Before characterizing the precise impact of start position on the spatial distribution of fixations, we consider the temporal properties of the eye movements and fixations.

#### Eye Movements for Center Start Position are Qualitatively Different From Other Start Positions

**Figure 5:**
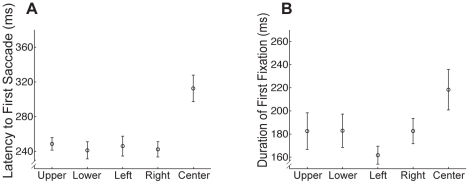
Impact of start position on timing of initial saccades and fixations for upright faces. (**a**) Average latency to first saccade by start position. Note the longer delay between face onset and the first saccade for the center compared to peripheral start positions. (**b**) Average duration of first fixation by start position. Note the longer fixation duration for the center compared to peripheral start positions. All error bars indicate the between-subjects standard error.

As noted earlier, one of the concerns about the commonly used center start position is that observers could be sampling information about the face even before making any eye movements. To investigate the possibility that information is sampled at center start position even before a saccade is made, we analyzed the latency to the first saccade i.e. the time between the onset of the face and the first saccade (Figure 5a). A one-way repeated measures ANOVA revealed a highly significant effect of Start Position (F(4,76) = 18.95, p<0.001). Paired comparisons (Bonferroni corrected) revealed that latencies did not vary across the four peripheral start locations (all p>0.38), but the latency to first saccade for the center start location was significantly longer than every one of the other start positions (all t>5.33, p<0.001). The increased time to make an eye movement away from the initial fixation position strongly suggests that there is some increased face processing at the center start position even before any eye movements have occurred.

Given this difference in saccade latency between center and peripheral start locations we further examined the duration of the first fixation following this initial saccade (Figure 5b). Two participants evidenced average durations less than 2.5 standard deviations from the group mean, and were excluded from this analysis. However, the overall results do not change with the inclusion of these participants. We observed the same pattern as for the initial saccade with longer fixation duration for the center start position compared with the others. A one-way repeated measures ANOVA revealed a significant effect of Start Position (F(4,68) = 3.07, p<0.05).

Taken together, these results suggest that, for the center start position, the experimenter and not the participant determine the initial information sampled. More generally, given the qualitative differences in the timing of eye movements between central and peripheral start positions, central and peripheral starting positions cannot be directly compared, and we focus in the rest of our analyses on the four peripheral start positions only.

#### First Fixation is Qualitatively Different From Later Fixations

**Figure 6:**
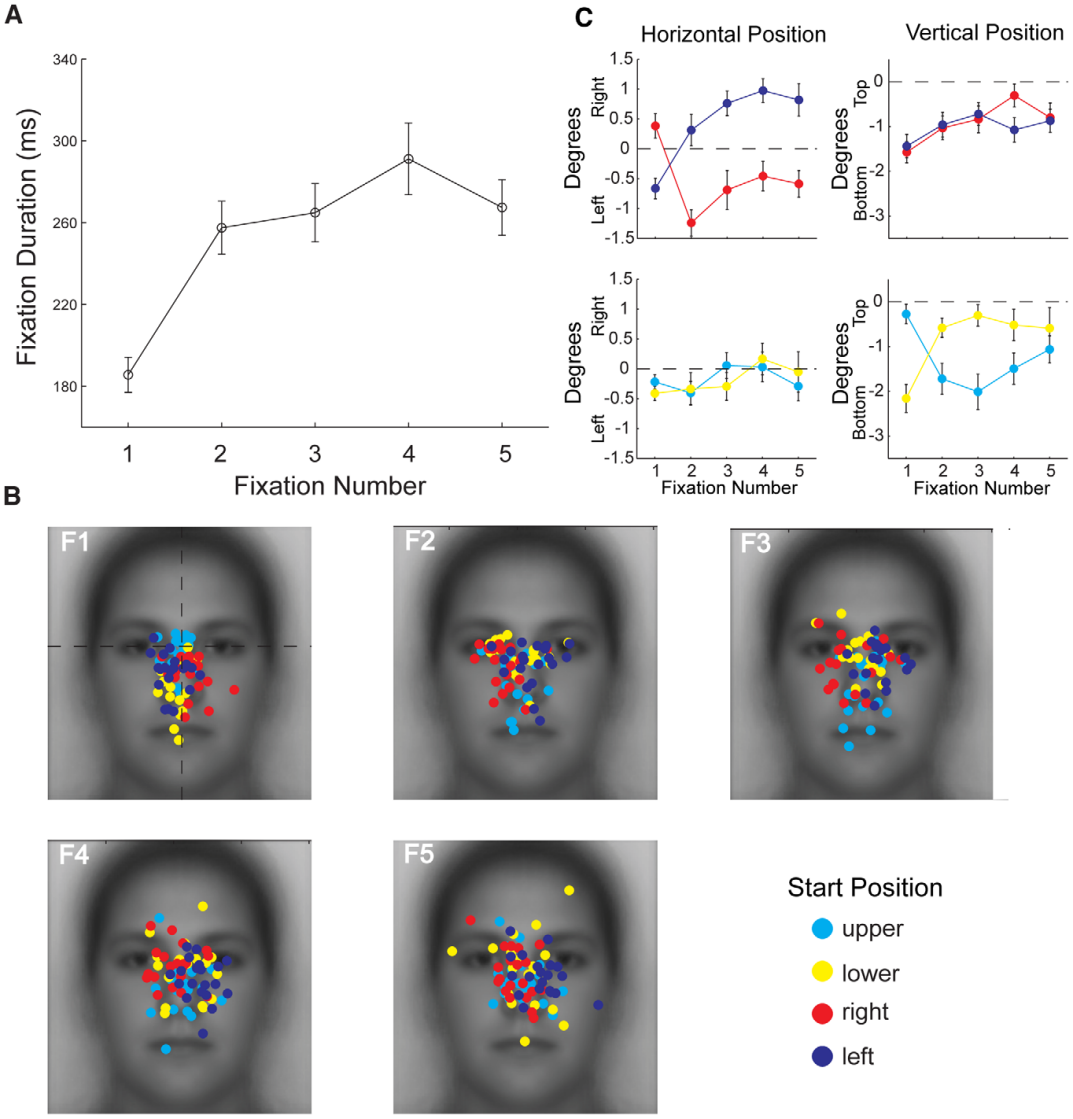
Evolution of fixations over ordinal number for upright faces. (**a**) Average duration of each ordinal fixation. Note the much shorter duration of the first than subsequent fixations. (**b**) Distribution of individual participants' fixation locations broken down by start position for each ordinal fixation (F1–F5). Fixation locations for the first fixation were generally toward the center of the face, but with a relative tendency to fall closer to the start position. Fixation locations for subsequent fixations tended to fall on the side of the face opposite the start position. For example, on the first fixation, fixations for the left start position show a tendency to the left side of the face while those for the right start position show a tendency to the right side of the face. On subsequent fixations, these tendencies reverse with the right start position showing a tendency to the left side of the face and the left start position to the right side of the face. A similar effect can be observed for the upper and lower start positions. (**c**) Average locations from (b). The two left plots give the average horizontal position of fixations in degrees of visual angle relative to the midline of the face (dotted line). The two right plots give the average vertical position relative to the vertical position of the eyes (Figure 3b). Note the strong effect of the left and right start positions on horizontal but not vertical position (top panels) and the opposite effect for the upper and lower start positions (bottom panels). Error bars indicate the between-subjects standard error.

We found that first fixation was shorter than subsequent fixations for all peripheral start positions (Figure 6a). Two-way ANOVA on fixation duration with peripheral Start Position (Left, Right, Upper, Lower) and Fixation Number (1–5) as factors revealed a significant main effect of Fixation Number (F(4,68) = 20.57, p<0.001), and a main effect of Start Position (F(3,51) = 2.96, p<0.048) arising from slightly longer average fixation durations for the lower and left start positions. Paired comparisons (Bonferroni corrected) between durations for each Fixation Number collapsed across Start Position revealed that the first fixation was shorter in duration than the other fixations (all t>5.50, p<0.001) and that the other fixations did not differ from each other in duration (all p>0.1).

To investigate the evolution of the fixation patterns with the center start position excluded, we plotted for each combination of peripheral start position and ordinal fixation number the average fixation location for each individual participant (Figure 6b). To evaluate the effect of start position we considered the group average location of each fixation in the horizontal and vertical dimensions (Figure 6c). On the first fixation there was a clear effect of start position. The first fixations landed near the center of the face, regardless of peripheral start position, with a slight tendency toward the start position itself. In particular, the first fixations for the right start position were significantly to the right of the first fixations for the left start position (t = 4.10, p<0.001) but these fixations did not vary in vertical position (t = 0.53, p>0.1) (Figure 6b, 6c). The opposite was true for the upper and lower start positions, for which the first fixations for the upper start position were significantly higher than the first fixations for the lower start position (t = 5.28, p<0.001) but did not vary in horizontal position (t = 1.70, p>0.1). On subsequent fixations, there was also a clear effect of start position, but with a tendency toward the side of face opposite the start position. Thus, the second fixations for the right start position were significantly to the left of the second fixations for the left start position (t = 7.76, p<0.001), but again there was no difference in vertical position (t = 0.99, p>0.1). Similarly the second fixations for the upper start position were significantly lower than the second fixations for the lower start position (t = 3.87, p<0.001) with no difference in horizontal position (t = 0.45, p>0.1). This tendency toward parts of the face opposite the start position was maintained throughout fixations 2–5 for the left/right start positions (all t>4.74, p<0.001), but was slightly weaker for the upper/lower start positions and was significant on fixations 2–4 only (all t>2.43, p<0.05). Importantly, the general spatial patterns observed on average across the first five fixations (Figure 4) only emerged on the second fixation. Combined with the finding that the first fixation is relatively short, this result suggests that the location of the first fixation is very heavily constrained by start position and may reflect a generic scanning strategy rather than any face specific processing. In light of this, we excluded the first fixation from the remainder of our fixation analyses for upright faces.

#### Direct contrasts of fixation patterns between start positions

**Figure 7:**
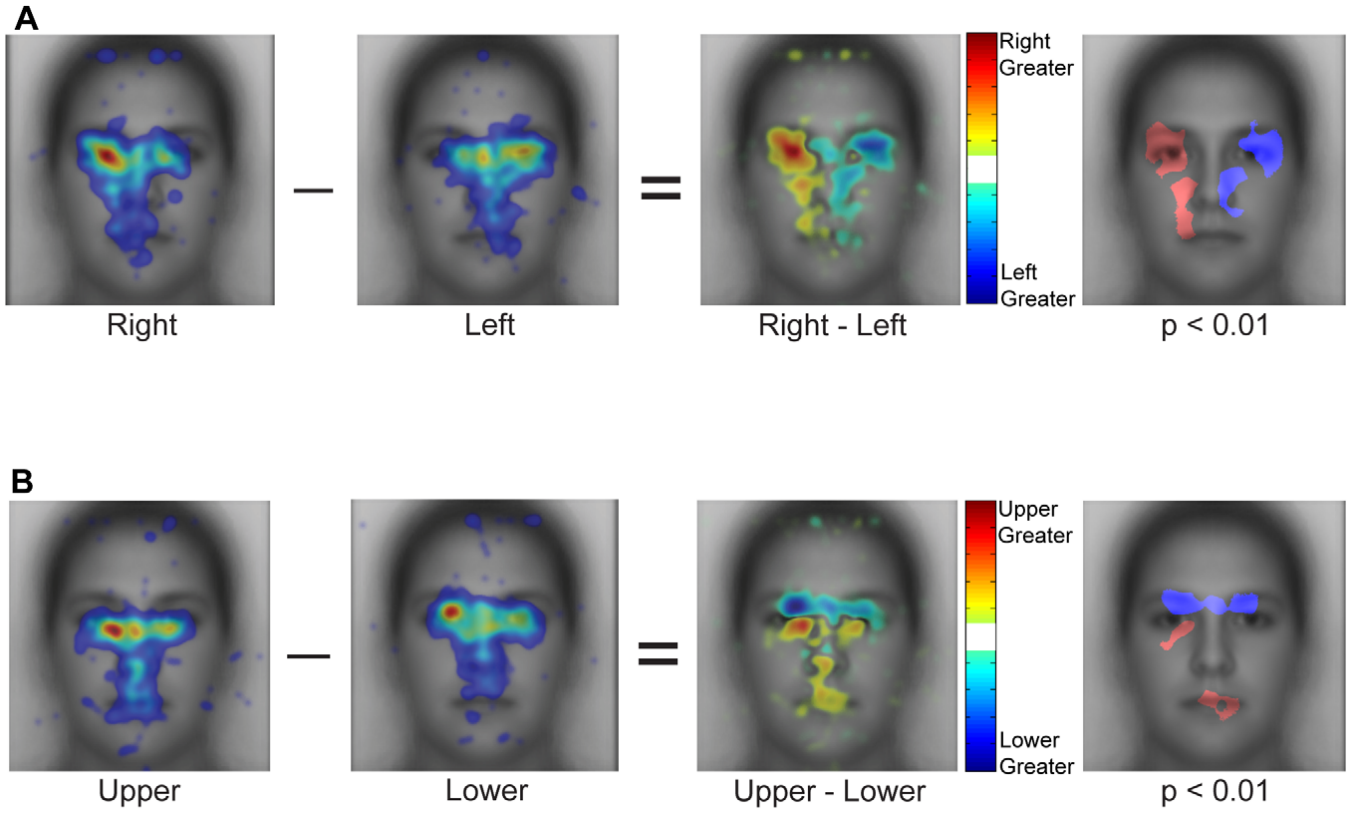
Direct comparison of spatial distributions of fixations for different start positions on upright faces. (**a**) Right vs. left start position. The first two panels are the raw spatial density maps for fixations 2–5. The third panel shows the subtraction of these spatial density maps. The fourth panel plots those locations where that difference was significant (p<0.01) according to a Monte Carlo permutation test, which assumed exchangeability of fixations across contrasted start positions for each ordinal fixation. The map was cluster corrected (cluster threshold p<0.05, see methods). Note the significant advantage for the side of the face opposite the start position. (**b**) Same as (a) but for the upper and lower start positions. Note again the strong and significant advantage for the side of the face opposite the start position. Fixation density in the raw heatmaps is indicated using a colorscale from zero to the maximum density value observed across the heatmaps for start position, with zero being transparent. The difference in fixation density in contrast heatmaps is indicated using a colorscale from plus to minus the largest absolute difference observed across start position contrast maps.

To characterize the effect of start position more precisely we conducted direct comparisons of the fixation patterns observed for the peripheral start positions. We conducted subtractions of the spatial density maps and computed statistical significance using Monte Carlo permutation tests (see Methods). For these analyses we used only fixations 2–5. Here, we focus on the comparison of left and right start positions (Figure 7a) and upper and lower start positions (Figure 7b). Note that in the spatial density maps for each start position in Figure 7 (with the first fixation removed), the profiles are more distinct than those shown earlier in Figure 4 with a weaker spatial density toward the center of the face.

Contrasting right versus left start position reveals a general advantage for the opposite side of the face (Figure 7a). The direct subtraction of these results reveals a symmetrical pattern with each start position showing a relative advantage for the opposite side of the face, primarily around the eyes but extending onto lower parts of the face as well. Thus, the right start position showed a relative advantage for the left eye while the left start position showed a relative advantage for the right eye.

A similar relative advantage for the opposite side of the face is also clear in the direct comparison of upper and lower start positions (Figure 7b). The upper start position showed a relative advantage for the mouth and nose while the lower start position showed a relative advantage for the upper part of the eye region bilaterally. Note that even though the eye region was fixated substantially with both upper and lower start positions, the precise locations of those fixations differed, with fixations for the upper start location predominantly below the eyes and fixations for the lower start position predominantly above the eyes.

In summary, fixation patterns for upright faces were highly influenced by start position. Most strikingly the often-reported advantage for the left side of the face was abolished for the left start position, which evidenced a right side advantage. This effect of start position was present even on the fifth fixation. The central start position, commonly used in prior studies [11], [14], [23], evidenced a longer latency to first saccade and first fixation duration than peripheral start positions suggesting that information is being sampled substantially even before the first saccade and highlights the strong potential biases likely introduced by the use of this start position. Regardless of start position, the first fixation was significantly shorter than the subsequent fixations and heavily impacted by start position. Finally, in general, fixations tended to fall on the opposite side of the face to the start position.

### Fixation Patterns: Inverted Faces

**Figure 8:**
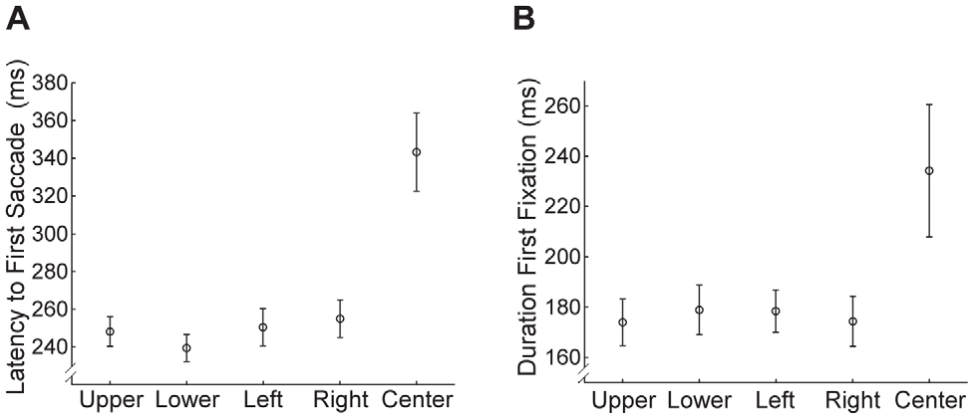
Impact of start position on timing of initial saccades and fixations for inverted faces. (**a**) Latency to first saccade. Note that the effect of start position was similar to that observed for upright faces with a longer latency for center compared to peripheral start positions (Figure 5a). (**b**) Duration of the first fixation. Again that the effect of start position was similar to that observed for upright faces with a longer fixation duration for center compared to peripheral start positions. All error bars indicate the between-subjects standard error.

We conducted the same series of analyses on inverted faces as we did for upright faces, finding very similar effects of start position and ordinal fixation number. As with upright faces, latency to first saccade (Figure 8a) was dependent on start position (F(4,76) = 23.903, p<0.001). Paired comparisons (Bonferroni corrected) revealed that the latency to first saccade for the center start position was significantly longer than any of the other start positions (all t>4.77, p<0.001), and there were no significant differences between latencies for the non-center start positions (all p>0.05). Further, as for upright faces, the duration of the first fixation was similarly dependent on start position (Figure 8b) (F(4,68) = 4.53, p<0.01) with longer first fixation durations for the center compared with peripheral start positions.

To directly test the effect of inversion on the temporal properties of the eye movements we ran a series of ANOVAs including Orientation as a factor. For latency to first saccade, as expected, there was a main effect of Start Position (F(4,76) = 34.98, p<0.001). In addition there was a main effect of Orientation (F(1,19) = 4.73, p = .042), arising from slightly longer latencies for inverted than upright faces, but there were no interactions involving Orientation (p>0.1). Thus, inversion had little impact on the overall effect of start position. An identical ANOVA on the duration of the first fixation (Figure 8b) revealed a main effect of Start Position only (F(4,68) = 7.83, p<0.001), reflecting the longer duration of the first fixation for the center start position, and no effects involving Orientation (p>0.1).

**Figure 9:**
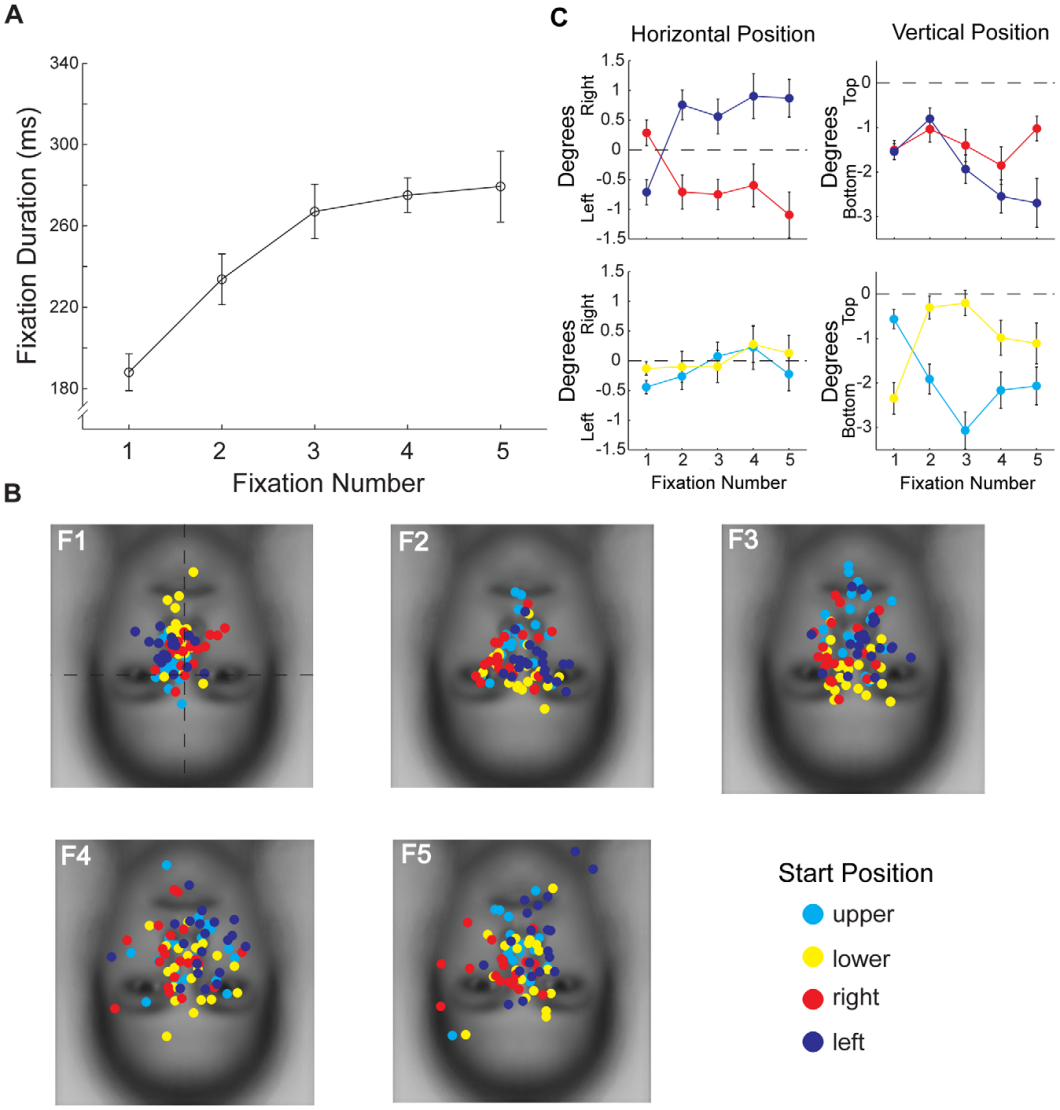
Evolution of fixations over ordinal number for inverted faces. (**a**) Average duration of each ordinal fixation. Note the much shorter duration of the first than subsequent fixations as was observed for upright faces. (**b**) Distribution of fixation locations across individual participants broken down by start position for each ordinal fixation (F1–F5). As for upright faces, fixation locations for the first fixation were generally toward the center of the face, but with a relative tendency to fall closer to the start position. Subsequent fixations locations tended to fall on the side of the face opposite the start position just as for upright faces. Note that all start positions are defined relative to the face. (**c**) Average locations from (b). Note the similar effects to those shown in Figure 6c. Error bars indicate the between-subjects standard error.

Focusing on the peripheral start positions only, an ANOVA on the fixation durations for inverted faces (Figure 9a) with Fixation Number and Start Position revealed a main effect of Fixation Number only (F(3,51) = 29.90, p<0.001). As with upright faces, the duration of the first fixation was shorter than fixations 2–5 (all t>5.18, p<0.001). However, unlike upright faces, the duration of the second fixation was also shorter than fixations 3–5 (all t>3.46, p<0.01). To directly compare upright and inverted faces, fixation durations were entered into an ANOVA with Fixation Number and Orientation as factors, revealing a main effect Fixation Number only (F(4,68) = 25.41, p<0.001) and no main effects or interactions involving Orientation (p<0.05). Thus, inversion had minimal impact on the different fixation durations for the first five fixations.

Plotting the average fixation location for each combination of peripheral start position and fixation number in each individual participant revealed very similar effects to those observed for upright faces. First fixations landed near the center of the face with a tendency toward the start position (Figure 9b). Thus, first fixations for the left start position were significantly to the left of those for the right start position (t = 3.09, p<0.01), and first fixations for the lower start position were significantly lower than first fixations for the upper start position (t = 4.98, p<0.001). As with upright faces, the central tendencies for each start position switched to the side of the face opposite the start position on the second and subsequent fixations. The horizontal difference in fixation location between the left and right start positions was significant for fixations 2–5 (all t>3.46, p<0.01), and the vertical difference between the upper and lower start positions was significant on fixations 2–4 (all t>3.60, p<0.002) (Figure 9c).

**Figure 10:**
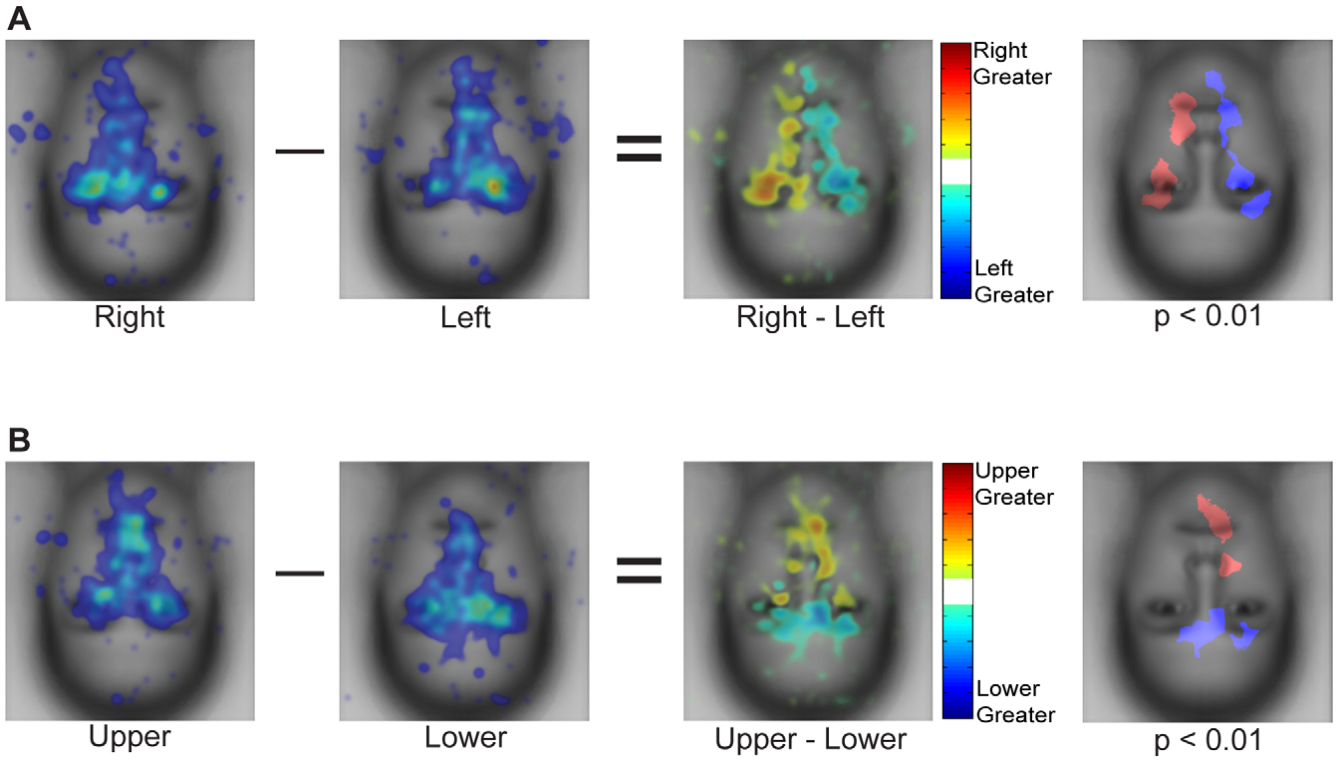
Direct comparison of spatial distributions of fixations for different start positions on inverted faces. (**a**) Contrast between right and left start positions. All conventions are the same as in Figure 7. Note the symmetrical advantage for the side of face opposite the start position as with upright faces. (**b**) Contrast between upper and lower start positions. Note, again, the advantage for the side of the face opposite the start position.

Direct comparison of the spatial density maps for the right and left start positions across fixations 2–5 revealed a similar pattern to that observed for upright faces, with each start position showing a relative advantage toward the opposite side of the face (Figure 10a). Similarly, comparison between upper and lower start positions revealed a relative advantage for the right side of the mouth and nose regions for the upper start position and toward the upper central part of the eye region for the lower start position (Figure 10b).

Overall, with inverted faces, we saw very similar effects of start position to those observed with upright faces. There was an increased latency to first saccade for the center compared with non-center start positions, a shorter first fixation, a general advantage for the upper parts of the face and a strong effect of start position with a relative advantage for the opposite side of the face for the non-center start positions.

#### Spatial density of fixations: upright versus inverted faces

**Figure 11:**
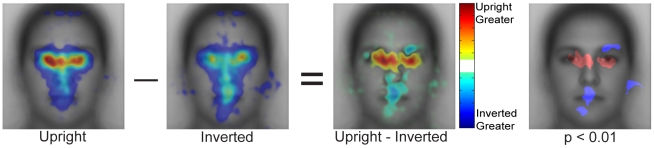
Direct comparison of spatial distributions of fixations for upright and inverted faces. The first two panels show the spatial density of fixations averaged across the peripheral start positions for upright and inverted faces, respectively. Note that there is greater variability in the location of fixations across the internal features for inverted than upright faces, but that the same general pattern holds. The third panel shows the subtraction of the first two panels and the fourth panel shows statistically significant differences. Overall, there are relatively more fixations to the eye region for upright compared to inverted faces and relatively fewer fixations to the mouth region. Fixation density in the raw heatmaps is indicated using a colorscale from zero to the maximum density value observed across the heatmaps pooling the peripheral start positions, with zero being transparent. The difference in fixation density in contrast heatmaps is indicated using a colorscale from plus to minus the largest absolute difference observed in the contrast map.

So far we have demonstrated similar effects of start position on upright and inverted faces, but have not directly compared the spatial density of fixations for upright versus inverted faces. A prior study [10] reported that for upright faces people make relatively more fixations to the eyes and relatively fewer fixations to the mouth region compared with inverted faces (but see [20], [38]). Comparison of Figures 7 and 10 shows that the overall spatial envelope of fixations is quite similar between upright and inverted faces. However, the relative advantage for the eye regions over the mouth regions is weaker for inverted compared with upright faces. To directly compare the pattern of eye movements for upright and inverted faces, we first contrasted the patterns of fixations averaged across start position, but excluding the first fixation and the central start position (Figure 11). While this difference reveals more fixations to the eye region for upright faces and more fixations to the mouth region for inverted faces it is important to note that there are some parts of the eye region that show a relative advantage for inverted faces. In particular, there are relatively more fixations to the upper part of the right eye for inverted faces compared with upright faces.

**Figure 12:**
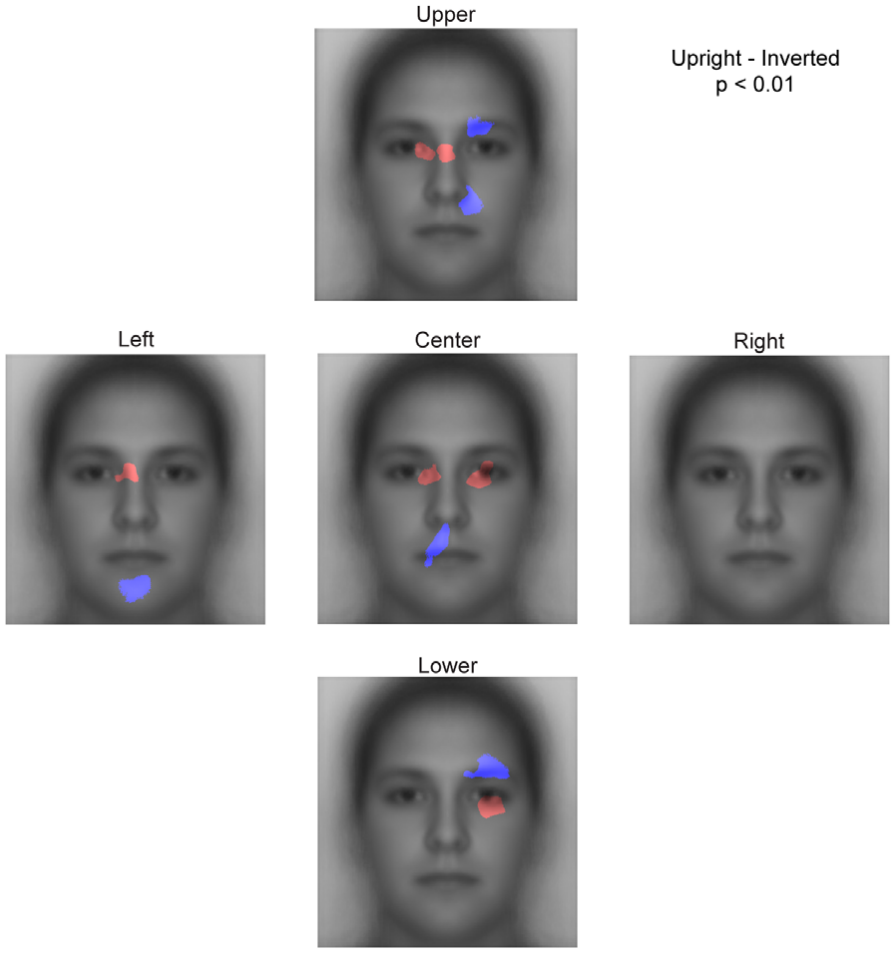
Impact of start position on the comparison of upright and inverted faces. Statistically thresholded maps for the contrast between upright and inverted faces by start position. Regions with p<0.01 significance for upright faces are shown in red and those for inverted faces are shown in blue. At a coarse scale, the difference is consistent with more fixations to the eyes in upright and toward the lower part of the face in inverted. However, the precise location and extent, particularly of which part of the lower face accrues more fixations in inverted and which part of the eye region accrues more fixations in upright varies with start position.

However, as we have shown above, there is a large effect of start position even for inverted faces. This effect of start position on both upright and inverted faces must inevitably affect the contrast of upright and inverted faces. Breaking down the contrast by start position, we find that while upright faces do seem to have relatively more fixations to the eyes and inverted faces relatively more fixations to the mouth region, the precise differences between upright and inverted are dependent on start position (Figure 12). For example, for both the upper and lower start positions, inverted faces have relatively more fixations above the right eye. This is not observed for the center and left start positions. Similarly, while the lower start position shows a relative advantage for inverted faces in the center of the mouth, for other start positions the advantage for inverted faces is on the chin or closer to the nose. These findings suggest that while the coarse difference between upright and inverted faces is similar across start positions (more fixations to the eye region for upright, more fixations to the mouth region for inverted), the specific locations of the difference in fixation pattern between upright and inverted faces vary as a function of start position.

Thus, fixation patterns for upright and inverted faces both show a relative advantage for the upper over the lower part of the face. At a coarse level, upright faces show relatively more fixations to the eye region and inverted faces to the mouth region consistent with at least one prior report [10]. However, the fine-scale differences in fixations between upright and inverted faces are dependent on start position.

### Fixation Patterns: Study Versus Test

So far we have only considered data from the study phase of the experiment, when viewing of the faces was relatively unconstrained. In the test phase of the experiment, faces were only presented for 1 second each and participants had to judge immediately whether they recognized the face or not. Participants made fewer fixations during face presentation in the test phase, but the basic patterns of fixations and effects of start position were very similar to the study phase.

**Figure 13:**
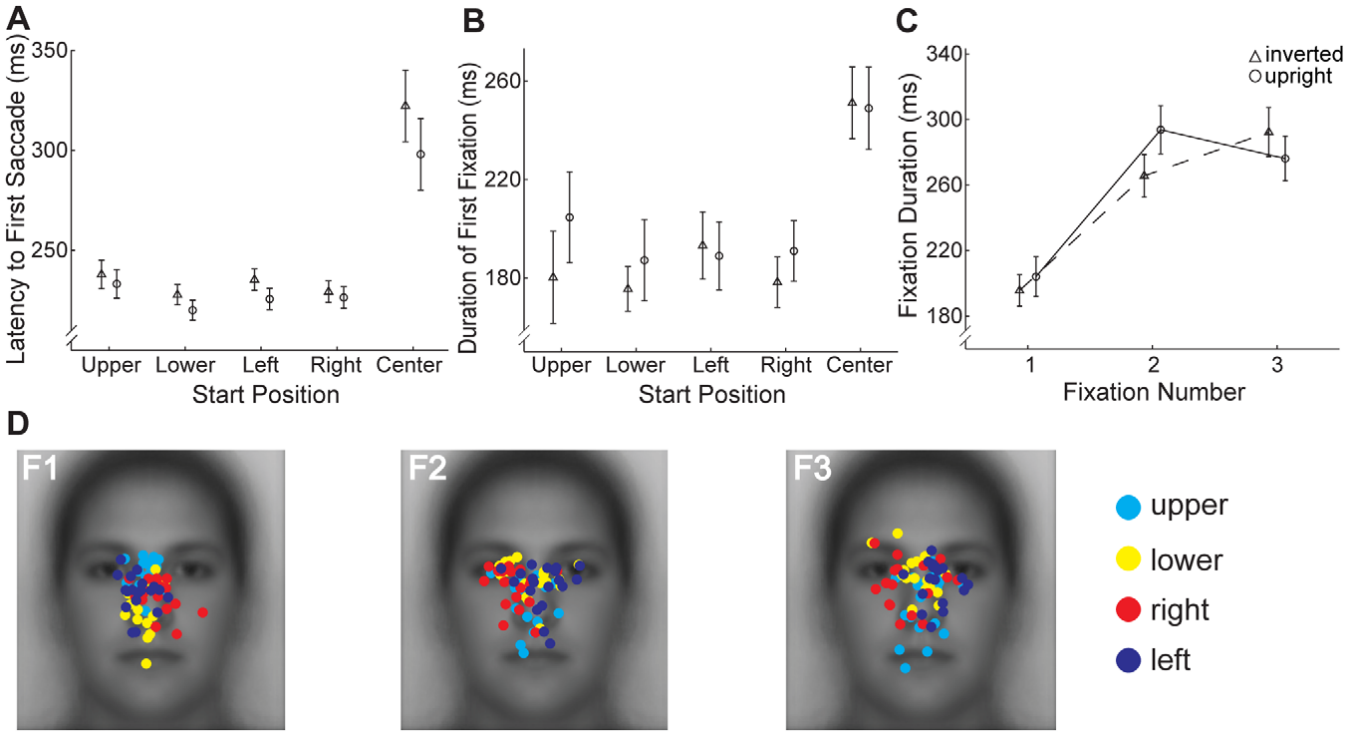
Analysis of fixations during the test phase. (**a**) Latency to first saccade by start position for upright and inverted faces. As for the study phase, there was a longer latency for the center start position compared with the peripheral start positions. (**b**) Duration of first fixation by start position for upright and inverted faces. Note the longer duration for the center start position, as observed during the study phase. (**c**) Duration of the first three fixations for the peripheral start locations for both upright and inverted faces. As for the study phase, the first fixation was significantly shorter than the subsequent fixations. All error bars indicate the between-subjects error. (**d**) Distribution of individual participants' fixation locations for upright faces broken down by start position for each fixation number (F1–F3). The same pattern was observed as during the study phase with first fixation close to the center of the face and subsequent fixations landing on the opposite side of the face to the start position.

In particular, the pattern of Start Position timing differences for the first saccade and subsequent fixations were nearly identical in study and test for both upright and inverted faces (Figure 13a–b). An ANOVA on latency to first saccade with Phase (Study, Test), Start Position, and Orientation as within-subject factors revealed a main effect of Start Position (F(4,76)>55.40, p<0.001), reflecting the longer latency for the center start position, and a main effect of Orientation (F(1,19)>8.82, p = 0.008), reflecting slightly longer latencies to the first saccade for inverted than upright faces. There was also a main effect of Phase (F(1,19)>21.12, p<0.001), and an interaction of Orientation and Start Position (F(4,76)>2.70, p = 0.037). An identical ANOVA on the duration of the first fixation also revealed a main effect of Start Position (F(4,60) = 12.79, p<0.001), again reflecting the longer duration for the center start position, but no other significant effects. Finally, an ANOVA on fixation duration across the peripheral start positions (Figure 13c) with Phase, Fixation Number (1,2,3), and Orientation revealed a main effect of Fixation Number (F(2,32) = 47.52, p<0.001), reflecting the shorter duration of the first fixation, and Phase (F(1,16) = 6.47, p<0.05) reflecting longer fixations during test than study. There was also an interaction between Orientation and Fixation Number (F(2,32) = 4.21, p<0.05), reflecting shorter 2nd fixations for inverted than upright faces. Thus, overall, the temporal characteristics of the eye movements we observed during study were very similar during test.

The general pattern of fixations observed in test was also very similar to that observed in study (Figure 13d). In general, the first fixation showed a strong tendency toward the side of the face closest to the start position, while the subsequent fixations showed a strong tendency toward the other side. Thus, the pattern of fixations observed in test were very similar to those observed in study, with the exception of there being fewer of them due to the restricted viewing time.

## Discussion

We investigated the effect of start position on the pattern of fixations observed when people view upright and inverted faces. Consistent with at least one previous study [10], we found greater consistency and more fixations on the eye region and less on the mouth region for upright compared with inverted faces, possibly reflecting differences in cognitive processing. However, we also found that start position, a non-stimulus, non-task factor, has a large impact on the location of fixations throughout the first five fixations of face viewing. In addition, we found that i) the center start position was qualitatively different from other start positions, with longer initial saccade latencies suggesting significant processing of facial information even prior to the first eye movement, and ii) for all start positions, the first fixation was qualitatively different from subsequent fixations with a shorter duration and a different spatial distribution of fixations. These effects were observed for both upright and inverted faces and during both study and test phases, suggesting they reflect basic properties of scanning eye movements. Taken together, the temporal and spatial effects of start position demonstrate that the absolute locations of fixations during face processing are strongly influenced by factors beyond stimuli and task, possibly reflecting influences of visuomotor effects or simple scanning strategies. Critically, our results suggest that previously reported fixation patterns based on a single start position or the average across multiple start positions may not accurately reflect the information used in face processing.

Considering first the peripheral start positions, each strongly influenced the pattern of fixations observed. First fixations landed near the center of face with a slight tendency toward the start position (see also [44]). On subsequent fixations this tendency flipped, and a strong tendency for fixations to land on the side of the face opposite the start position emerged. Combined with the much shorter duration of the first compared to subsequent fixations, this suggests that the location of the first fixation is highly dependent on the start position and may reflect a simple initial localizing saccade. [44] It has previously been suggested [28] that the location of the first fixation on faces may reflect the center-of-gravity effect or the tendency of saccades to land at the center of objects [29], [30]. Note, however, that Bindemann and colleagues [28] pooled their data across start positions making it unclear to what extent the effect they observed reflects a center-of-gravity effect or an artifact of averaging. Arguably, the location of the first fixations in our data could reflect a center-of-gravity effect combined with a saccadic undershoot [31], [32], causing the maintained tendency toward the start position. However, this explanation is insufficient to explain the persistence of the differences in fixation patterns across the subsequent fixations. Further, and most importantly, in terms of information use it is the precise fixation location that is critical, as it is the point of highest acuity, not the presumptive target of any saccade.

Following the initial fixation, we observed a subsequent strong bias to the opposite side of the face. Strikingly, the previously reported tendency to fixate the left over the right side of the face [6], [43], [45], [46], which has been assumed to reflect a left-side bias and right hemisphere dominance in face perception [45], [47], [48], was reversed in our data for the left start position (but present for other start positions). This pattern of results is consistent with a naive sampling strategy in which the observer simply fixates high contrast facial features as far from those that have already been sampled as possible, increasing information gain. However, recent studies suggest that simple information gain alone may not drive eye movements. For example, in a study of eye movements to simple geometric contours [44], eye movements were better characterized as reducing local uncertainty, not maximizing total information gained. Further, when peripheral background information is masked or blurred, eye movements tend to follow intact information near the fovea, rather then projecting into unseen regions, counter to the predictions of information gain [49].

Previous studies adopting multiple off-face starting positions [6], [7], [21], [28] have varied the position of the face, with an initial fixation at the center of the screen, we used a constant face position at screen center and varied the location of the initial fixation. We chose this approach so that any differences in eye movements could not be ascribed to effects caused by, for example, the varying location of the edge of the screen with respect to the face. While the general position of the face was therefore predictable in our study, in prior studies the position was unpredictable preventing participants from planning any eye movements until the face had actually appeared on the screen. Although, this predictability may have contributed to the effect of start position we observed, we would still expect an effect of start position when face position is unpredictable since there is still variation in the relative location of facial features with respect to the start position.

Despite the large differences in fixation patterns caused by varying start position, behavioral performance was very similar across start positions. Even, for the left start position when participants showed a preference for the right rather than the left side of the face, there was no impact on behavioral performance. These results suggest two main possibilities, which are not mutually exclusive. First, the processing of facial information may not be tightly restricted to the fovea, and the specific fixation locations only weakly linked to the information being extracted [50]. For example, fixation locations near the eyes for the upper start position tended to land below the eyes, whereas those for the lower start position tended to land just above the eyes. In both cases the participants may be extracting similar information from the eyes. Second, a recent study, in which starting positions were above and below the face, reported that two fixations only were sufficient for participants to achieve optimal recognition performance [6]. This finding implies that only a very limited subset of the information foveated during free scanning is necessary for recognition. Given the large variation in fixation patterns we observed over the first two fixations across start positions, this finding implies many different fixation patterns are capable of supporting face recognition. These considerations highlight how important it is to directly relate eye movement patterns with behavioral performance to ascertain their significance (e.g. [6]).

**Figure 14:**
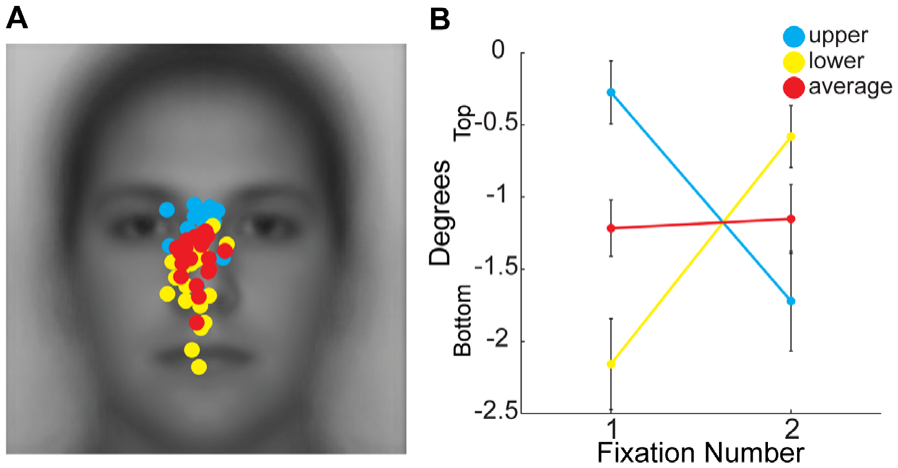
The problem of averaging across start positions. (**a**) First fixation locations across participants for the upper (blue) and lower (yellow) start positions for upright faces are replotted from Figure 6. The average location across these two start positions is plotted for each individual subject in red. Note that this averaging causes a regression to the center of face and obscures the tendency to fixate the side of face closest to the start location. Importantly, there is very little overlap in the distributions of fixation locations for the upper and lower start positions. (**b**) Average fixation locations, relative to the position of the eyes, in the vertical dimension for the upper and lower start positions for the first two fixations replotted from Figure 6. The average vertical location between these two start positions is plotted in red. Note that the average completely obscures the large shift in vertical bias between the first and second fixation. Error bars indicate the between-subjects error.

Based on the average fixation locations over the first two fixations, Hsiao and Cottrell concluded that fixations near the center of the nose were optimal for recognition [6]. However, it is important to note that they averaged data from two start positions roughly corresponding to our upper and lower start positions. A close look at our data shows that for upright faces while the average fixation location for both the first and second fixation indeed land near the center of the nose, there are large differences in fixation patterns for these two start positions (Figure 14a). First, very few fixations actually land in the location identified by the averaging and thus suggestions that either information available at this location or that fixations to this location are optimal are tenuous. Second, the locations of fixations for the upper and lower start position reverse across the first two fixations. On the first fixation, the fixations for the upper start position are near the bridge of the nose, while those for the lower start position are near the tip of the nose. However, on the second fixation, these distributions completely switch. This striking difference is completely obscured by averaging across start positions (Figure 14b).

The problem of biases introduced by specific start positions is perhaps most serious for the frequently used center start position. In our data, the delayed initial saccade latency and longer initial fixation both suggest that substantial face processing begins at the initial presentation of the face. Despite its wide use in the literature (e.g. [11], [14], [23]), this start position clearly introduces processing of facial information which is entirely dependent on the experimenter's choice of the position of the fixation cross on the face rather than on anything particular to the stimulus and task. Unfortunately, there is no easy solution to this problem as excluding the fixation prior to the first saccade simultaneously excludes the processing occurring during that fixation, and including the first fixation blends the experimental bias into the analysis. The difference in the temporal dynamics between the center and peripheral start locations also renders direct comparisons either within or across studies very difficult. It is impossible to reasonably equate the first fixation of a center start position trial with one that used a peripheral start position. This is not to suggest that the center start position should never be used, rather it can only provide useful information based on comparisons between conditions that used only the center start position and if it is used any interpretation of fixation data must take into account the information that is likely extracted prior to the first saccade.

Consistent with differences in cognitive processing for upright and inverted faces and data from at least one prior eye tracking study [10], we did observe differences in fixations to upright and inverted faces. While the distributions of fixations for upright and inverted faces were both largely confined to the internal facial features with a similar spatial envelope, for inverted faces there were *relatively* fewer fixations on the eye region and *relatively* more on the lower part of the face. However, the *absolute* tendency toward the upper part of the face was not completely eliminated under inversion. These differences in fixations between upright and inverted faces may contribute to the poorer behavioral performance for inverted faces. Further, while the course effects were similar, the precise location of the differences between upright and inverted faces differed by start position.

In contrast to our findings and those of Barton and colleagues [10], two other studies of fixations to upright and inverted faces reported little or no effect of orientation [20], [38]. There are a number of possible explanations for this discrepancy. First, differences in experimental design may be important. For example, Rodger and colleagues did observe differential fixation patterns between upright and inverted faces that are qualitatively similar to those we found (compare our Figure 11 with the data from Western Caucasian participants in their Figure 5). These differences did not reach significance in their analyses, but this may largely be an issue of power as inversion was a between-subjects factor in their study but within-subjects in ours. Williams and Henderson [20] did test inversion within-subject, but they principally used AOI analyses, which are strongly impacted by the precise definition of the AOI borders (for discussion of this issue see, for example, [38], [42]). Second, and more generally, the strong effect of start position we observed suggests that other non-stimulus, non-task factors may have also influenced fixations during face processing. For example, in comparing our findings with those of Rodger and colleagues [38] and Williams and Henderson [20] design differences such as the size of the faces or the distance of the initial fixation from the faces may also be having an impact. To the extent that non-stimulus, non-task factors affect fixation patterns, comparisons between studies and generalizations of findings are difficult to make.

### Implications for Eye tracking Research

Our findings have important implications for the use of fixation patterns as an index of information processing and for the design of eye tracking studies. Our study focused on face processing, but our findings likely extend to use of visual fixations in other stimulus domains. Most importantly, our results demonstrate that interpreting the absolute location of fixations is extremely difficult. This caveat, particularly as it relates to potential visuomotor effects on fixation locations, is already well understood in the reading literature (e.g. [51], [52]) but seems to be largely ignored in studies of eye movements to faces. Eye tracking studies must take into account even the simple case of variation in start position, as absolute fixation locations may reflect tendencies introduced by the start position as much as the stimuli and the task. In particular, given the processing that likely occurs even prior to the first saccade, it is preferential to avoid using a center start position. Given the wide variation in fixation patterns observed between start positions, multiple start positions should be used and direct comparisons should be made between these start positions. Importantly, our findings suggest that simply averaging data across start positions will tend to artificially regress fixation locations toward the center of the face. Importantly, we do not mean to suggest that fixation patterns cannot provide useful information about visual processing, nor that eye movements are epiphenomenal. In fact, it has been demonstrated that the ability to freely make eye movements improves discrimination performance for faces [8], [9]. Furthermore, prior studies demonstrating differences in fixation patterns between groups or between conditions are not incorrect. The problem is in interpreting what the differences in absolute location actually mean with respect to the information utilized. Additionally, our study shows that the non-stimulus, non-task factors imposed in experiments can drastically influence eye-movement patterns, and so the eye-movements observed in previous studies may not reflect ecological eye-movements as closely as has been assumed. Alternative approaches, such as gaze-contingent paradigms (e.g. [49], [50], [53]) in which the information available at each fixation is systematically controlled, may help overcome some of these difficulties. The addition of converging evidence from other paradigms assessing information use, such as Bubbles [54] or reverse correlation analyses [55], [56], would also strengthen any claims. In sum, what we are suggesting is that the effect of experimental procedure must be carefully considered and controlled before making direct links between eye movement patterns and information use.
